# Retraction: Molecular Diagnosis of Primary Hyperoxaluria Type 1 and Distal Renal Tubular Acidosis in Moroccan Patients With Nephrolithiasis and/or Nephrocalcinosis

**DOI:** 10.7759/cureus.r62

**Published:** 2022-10-10

**Authors:** Abdouss Fatima, Ahakoud Mohamed, Hida Moustapha, Ouldim Karim

**Affiliations:** 1 Biomedical and Translational Research Laboratory, Faculty of Medicine and Pharmacy, Sidi Mohamed Ben Abdellah University, Fez, MAR; 2 Medical Genetics and Onco-genetics Laboratory, Central Laboratory of Medical Analysis, Hospital University Hassan II, Fez, MAR; 3 Pediatric Department, Mother and Child Hospital, Hospital University Hassan II, Fez, MAR

This article has been retracted due to unethical research practices and data manipulation, falsification and fabrication. The misconduct associated with this article was brought to the attention of the journal by senior faculty at Sidi Mohamed Ben Abdellah University and University Hospital Hassan II, who conducted a detailed investigation and provided the journal with the results, signed by the Dean and Vice Deans of faculty as well as the leaders of the departments involved, and the authors themselves.

As a result of the investigation, the following infractions were identified, confirmed and detailed in the letter received by the journal:

- First author Fatima Abdouss submitted the article without the knowledge or consent of her co-authors.
- Fatima Abdouss knowingly provided a false affiliation as she is no longer a member of the cited research laboratory (Biomedical and Translational Research Laboratory, Faculty of Medicine and Pharmacy, Sidi Mohamed Ben Abdellah University). Her registration in this laboratory as a doctoral student was not renewed in September 2021 due to a lack of progress in her research work and the presentation of false data.
- The article was submitted and published without the knowledge of the department heads cited as the source of the patient's data, Prof. Leila Bougnouch and Prof. Fatima Zohra Souilmi. Both had previously refused the submission of this article in 2021 after discovering it contains fraudulent data (false number of patients, false clinical and radiological data, several non-existent patients, false statistics, etc.).
- Prof. Tarik Sqalli Houssaini confirms that no patient from his Nephrology Department was included in the study, despite the article's claims.
- The patient consent or legal tutor consent for minors was not obtained despite Fatima Abdouss claiming otherwise in her submission.

This article has been retracted as a result of the aforementioned egregious academic fraud and misconduct by Fatima Abdouss. The journal is thankful for the detailed and swift response from the involved institution.

